# 选择性动脉灌注化疗联合靶向药物治疗非小细胞肺癌多发脑转移

**DOI:** 10.3779/j.issn.1009-3419.2012.05.11

**Published:** 2012-05-20

**Authors:** 金铎 李, 志 郭

**Affiliations:** 1 300060 天津，天津医科大学附属天津肿瘤医院介入治疗科，天津市肿瘤防治重点实验室 Department of Intervention, Tianjin Medical University Cancer Institute and Hospital, Key Laboratory of Cancer Prevention and Therapy, Tianjin 300060, China; 2 300060 天津，天津市环湖医院 Tianjin Huanhu Hospital, Tianjin 300060, China

**Keywords:** 肺肿瘤, 脑转移, 靶向药物, 动脉化疗, 介入治疗, 预后因素, Lung neoplasms, Brain metastases, Target drug, Arterial chemotherapy, Interventional therapy, Prognosis factor

## Abstract

**背景与目的:**

本研究旨在回顾性分析选择性动脉灌注化疗联合赖氨酸激酶抑制剂（tyrosine kinase inhibitor, TKI）药物治疗非小细胞肺癌（non-small cell lung cancer, NSCLC）多发脑转移的临床疗效及预后相关因素。

**方法:**

自2008年9月-2011年10月共入组31例诊断明确的非小细胞肺癌经CT或MRI证实多发脑转移瘤（> 3个）的患者，行选择性颅内动脉、支气管动脉及相关靶动脉化疗药物灌注化疗2个-6个周期，每周期间隔4周，同步或后续联合厄洛替尼、吉非替尼或埃克替尼治疗。介入治疗2个周期或联合靶向治疗后每4周应用CT和MRI对肿瘤进行疗效评价，直至肿瘤进展或发生不可耐受性化疗药物不良反应。

**结果:**

31例患者平均行3个周期介入治疗，随访时间4个-40.9个月，完全缓解5例（16.1%），部分缓解7例（22.6%），疾病稳定11例（35.5%），疾病进展8例（25.8%）。客观缓解率（objective response rate, ORR）为38.7%，疾病控制率（disease control rate, DCR）为74.2%。中位无进展生存期（progression free survival, PFS）为13.1个月，中位总生存期（overall survival, OS）为15.1个月。6个月的生存率为79%，1年生存率为61.1%，2年生存率为31.1%。分层分析显示PFS、OS与吸烟状态、病理类型、颅外转移情况、靶向药物应用时间、PS评分具有相关性；与性别、年龄、既往治疗情况、脑转移数目无明显相关。

**结论:**

选择性动脉灌注化疗联合靶向药物是治疗非小细胞肺癌多发脑转移安全有效的方法之一，吸烟状态、PS评分、肿瘤病理类型、颅外转移状况、靶向药物应用时间均可影响患者预后。

## Abstract (en)

**Background and objective:**

The aim of this study is to evaluate the efficacy of selected arterial infusion chemotherapy in treating non-small cell lung cancer (NSCLC) with multiple brain metastases and corresponding factors to influencing prognosis.

**Methods:**

From September 2008 to October 2011, a total of 31 patients of NSCLC with multiple brain metastases (≥3) received selected incranial, bronchial and corresponding target arterial infusion chemotherapy combined with EGFR-TKIs. Interventional treatment was performed every four weeks, two-six cycles with synchronized or sequential targeted drugs (erlotinib, gefitinib or icotinib). Follow-up CT and MRI were regularly finished at interval of four weeks after two cycles of interventional treatment were finished or during taking targeted drugs in order to evaluate efficacy of the therapy. The procedure was stopped for the tumor disease was worse or the patient could not tolerate the toxity of drugs any longer.

**Results:**

31 patients was performed two to six cycles of interventional therapy, 3cycles at average. Response assessment showed that 5 (16.1%) patients got a complete response (CR), 7 (22.6%) had a partial response (PR), 11 (35.5%) had a stable disease (SD) and 8 (25.8%) had a progressive disease (PD). The objective response rate (ORR) was 38.7%, and the disease control rate was 74.2%. The median progression free survival (PFS) and overall survival (OS) were 13.1 months and 15.1 months. The 6-month survival rate, one-year survival rate and two-year survival rate were 79%, 61.1%, and 31.1%, respectively. The patients' OS and PFS were influenced by smoking state, tumor pathology, extracranial metastases, period of targeted drug taking and performance status, not by sex, age, before therapy and the total of brain metastases.

**Conclusion:**

Selected arterial infusion chemotherapy with targeted drugs is one of the most effective and safe treatment to NSCLC with multiple brain metastases. Smoking status, tumor pathology, extracranial metastases, targeted drug taking and performance status are corresponding the patient's prognosis.

非小细胞肺癌（non-small cell lung cancer, NSCLC）是目前脑转移发生率最高的恶性肿瘤之一。文献^[1, 2]^报道初诊时即有16.3%-19.9%的NSCLC患者伴发脑转移，其中腺癌患者脑转移发生率高达43%，同时70%的NSCLC脑转移为多发病灶。相对于单发或少发脑转移，多发脑转移（> 3个）的NSCLC患者预后较差，目前标准的治疗方案（whole brain radiotherapy, WBRT）虽可使60%-80%患者的中枢神经系统症状得以缓解，神经系统功能得以改善，但中位生存期仅为3个-6个月，多数患者最终死于脑转移灶进展^[3]^。

虽NSCLC多发脑转移临床上常见，但专门针对此类患者的临床研究相对较少。我院从2008年9月-2011年10月收治初治或复治非小细胞肺癌多发脑转移患者，应用选择性颅内动脉、支气管动脉介入化疗联合靶向药物治疗31例，现将治疗效果及预后情况做以分析，以供临床治疗提供参考。

## 1 资料与方法

### 1.1 一般资料

入组患者符合如下标准：①经病理组织学证实原发灶为非小细胞肺癌；②经MRI或CT确诊为脑转移且脑转移瘤数目3个或3个以上；③存在至少1个经CT或MRI证实的可评估病灶；④经既往治疗病情进展或初治、拒绝行手术、放疗及静脉化疗的患者；⑤无严重的器官功能不全者；⑥患者自愿参加，并签署知情同意书。

### 1.2 治疗方法

入组患者均行选择性颅内动脉、支气管动脉及相关肋间动脉化疗药物灌注化疗，同步或后续应用靶向药物治疗。

介入治疗采用Seldinger穿刺技术，经一侧股动脉穿刺、插管，在数字减影机下导管选择至相关肿瘤供养动脉。针对颅内转移瘤，依据术前头CT或MRI检查，明确肿瘤病变部位，应用4F单弯导管选择至单侧或双侧颈内动脉及椎动脉造影，确认肿瘤供养动脉后，应用动脉输液泵以4 mL/min的输注速度依次将20%甘露醇20 mL以及经5%GS 20 mL-40 mL+鬼臼噻吩甙（VM-26）50 mg-100 mg、0.9%NaCl 20 mL-40 mL+尼莫司汀（ACNU）25 mg-50 mg和5%GS 20 mL-40 mL卡铂（CBP）50 mg-200 mg；对肺内原发病灶及颅外转移灶同时行支气管动脉及相关颅外转移灶靶动脉灌注化疗，用药为羟喜树碱（HCPT）20 mg-30 mg，卡铂（CBP）100 mg-200 mg，多西他赛（DOC）40 mg-60 mg或吉西他滨（GEM）400 mg-600 mg，经5%GS或0.9%NaCl稀释后经动脉输液泵缓注于靶动脉内；每周期间隔4周，平均完成2个-6个周期，待完成介入治疗2个周期后4周复查头颅强化MRI、胸CT、及相关颅外转移病灶检查，并进行疗效评价。在行介入治疗期间或介入治疗完成后应用表皮生长因子受体酪氨酸激酶抑制剂（epidermal growth factor receptor tyrosine kinase inhibitor, EGFR-TKI）类药物联合治疗，药物选择：吉非替尼250 mg qd，或厄洛替尼150 mg qd，或埃克替尼125 mg tid。患者服用靶向药物至出现不可耐受的不良反应或连续两次评价为疾病进展。介入治疗术后给予降低脑水肿，继续20%甘露醇250 mL静脉滴注，并根据肿瘤周围脑组织水肿情况及临床表现调整用药频次，并相应给予抗菌素抗肺感染及预防肺感染治疗。

### 1.3 疗效及不良反应评价标准

介入治疗2个周期后进行疗效评价，或联合靶向治疗每4周应用CT或MRI进行疗效评价。

#### 1.3.1 近期疗效评价

根据实体瘤疗效评价标准（RECIST）即可测量病灶最大直径总和之变化分为完全缓解（complete response, CR），即全部病灶消失并维持4周以上；部分缓解（partial response, PR），病灶缩小至少30%并维持4周以上；疾病稳定（stable disease, SD），介于部分缓解与疾病进展之间；疾病进展（progressive disease, PD），单发病灶直径扩大 > 20%或多发病灶增加 > 20%，或出现新病灶。客观缓解率（objective response rate, ORR）=CR+PR；疾病控制率（disease control rate, DCR）=CR+PR+SD。

#### 1.3.2 生存状况

无进展生存期（progression free survival, PFS）指患者首次治疗至出现疾病进展或末次随访时间。总生存期（overall survival, OS）指患者首次治疗至因任何原因死亡或末次随访日期的时间。

#### 1.3.3 药物不良反应评价

按照美国国立癌症研究所制订的通用药物毒性反应标准（National Cancer Institute Common Toxicity Criteria, NCI-CTC）3.0版分级标准，分为0级-4级，对治疗期间或随访期间出现的各种不良事件进行评价。

### 1.4 统计分析方法

研究数据采用SPSS 19.0软件进行分析。计数资料用构成比（%）或率（%）表示，生存分析采用*Kaplan-Meier*方法并进行*Log-rank*检验和*Cox*回归分析，以*P* < 0.05为差异有统计学意义。

## 2 结果

### 2.1 患者一般资料

自2008年9月-2011年10月共入组31例患者。其中，男性17例，女性14例，年龄31岁-77岁，中位年龄59岁。腺癌19例，鳞癌9例，大细胞癌1例，不能分型2例。患者体力状况（performance status, PS）评分，1分4例，2分14例，3分12例，4分1例。吸烟18例，不吸烟13例。初治20例，复治11例。脑转移总数332个（3个-45个），平均10.7个。颅外转移16例，无颅外转移15例。患者共接受2次-6次介入治疗，人均行介入治疗次数3次。口服厄洛替尼29例，吉非替尼1例，埃克替尼1例，平均服药时间为6.9个月。治疗期间无一例死亡。

### 2.2 临床疗效

本组患者均可评价疗效，其中CR 5例（16.1%），PR 7例（22.6%），SD 11例（35.5%），PD 8例（25.8%）。ORR为38.7%，DCR为74.2%。

### 2.3 生存分析

本组患者随访时间4个-40.9个月，中位随访时间为9个月，无失访病例。截止2012年2月1日死亡17例，中位PFS为13.1个月，中位OS为15.1个月。6个月的生存率为79%，1年生存率为61.1%，2年生存率为31.1%。分层分析显示PFS、OS与吸烟状态、病理类型、颅外转移情况、靶向药物应用时间、KPS评分具有相关性，与性别、年龄、既往治疗情况、脑转移数目无明显相关。但女性的中位PFS、OS分别为16个月和17.9个月，男性11.5个月和13.5个月，女性明显有生存优势但未达到统计学差异。

### 2.4 不良反应

本组病例不良反应情况：皮疹24例（77.4%）。贫血7例（22.6%），白细胞减少6例（19.3%），血小板减少4例（12.9%）。胃肠道反应5例（16.9%），上述不良反应均以1级-2级为主。其中1例发生间质性肺炎。未观察到严重的不良反应。

## 3 讨论

目前，非小细胞肺癌脑转移的治疗手段主要包括全脑放疗（WBRT）、立体定向放射治疗（stereotactic radiosurgery, SRS）、手术、靶向治疗和化疗等。然而目前WBRT仍是NSCLC多发脑转移的标准治疗方式，但远期疗效并不理想，同时研究显示并非所有的患者从中获益。Sundaresan等^[4]^回顾性研究显示24%的NSCLC多发脑转移患者在WBRT后4周内死亡，66%的PS > 2分的患者不能从中获益，其不能获益的发生率是PS≤2分患者的10倍。然而WBRT+化疗联合治疗的疗效也是有限的，最近的一项二期临床研究培美曲塞+顺铂6疗程后续贯WBRT一线治疗肺腺癌多发脑转移的颅内缓解率为41.9%，中位PFS为4个月，中位OS仅为7.4个月^[5]^。对于SRS主要应用于单发或是少发的脑转移肿瘤患者，目前转移灶≤3个、单个转移灶最大径≤3 cm疗效较好，逐渐成为脑转移瘤的标准治疗。

Serizawa等^[6]^根据多中心临床研究分析脑转移灶数目与疗效的关系，5个-10个脑转移灶同2个-4个脑转移灶的预后相当，将转移灶个数进一步细分，则3个-4个、5个-6个、7个-10个脑转移的中位OS分别为0.69年、0.59年及0.62年，患者的总生存期并没有明显延长，且对10个以上的脑转移灶的疗效未再分析。

选择性动脉灌注化疗采用Seldinger穿刺技术，经一侧股动脉穿刺、插管，在数字减影机下导管选择至相关肿瘤供养动脉，应用动脉输液泵通过导管将化疗药物经稀释后缓慢灌注相关靶动脉内。动脉灌注化疗由于将药物直接灌注肿瘤供养动脉内，未经静脉血液稀释及肝的首过清除效应，血药浓度高，使肿瘤组织内药物浓度明显高于静脉化疗，且经动脉泵缓注于动脉内每种化疗药物将持续15 min-30 min完成，从而充分提高化疗药物对肿瘤细胞的杀伤力，对于局部肿瘤达到有效的控制^[7-9]^。此外，由于局部给药，药物总剂量仅为全身化疗剂量的1/4-1/2，降低了化疗药物对全身各系统引起的不良反应，从而提高患者对化疗药物的耐受性，部分PS为3分的患者亦可从中获益。然而局部动脉灌注化疗对局部肿瘤有效，但远期生存并不理想，需联合全身抗肿瘤治疗可能达到更好的临床疗效。

吉非替尼、厄洛替尼、埃克替尼均是表皮生长因子受体酪氨酸激酶抑制剂。针对吉非替尼及厄洛替尼多项大型临床研究^[10, 11]^表明，显示在有选择的NSCLC治疗中较静脉化疗存在明显的优势，延缓疾病进展，预防症状恶化，维持PS以保证患者接受更多的治疗，延长患者的OS。埃克替尼（商品名称：凯美纳）是我国浙江贝达药业自主研发的第一个应用于NSCLC的靶向药物，其疗效不次于吉非替尼，有更好的安全性^[12, 13]^。

本组病例应用动脉灌注化疗联合靶向药物综合治疗后，既克服了单纯动脉灌注化疗的远期生存的不足又兼顾了单纯应用靶向药物对于KPS评分较低的患者近期疗效。本组病例主要以PS为2分-3分的患者为主（83.9%），可达到ORR为38.7%、DCR为74.2%的近期疗效。对于远期疗效，PS 2分的患者中位OS为15.2个月，3分达到9个月，明显延长了患者的总生存。本组病例副反应轻微，且未出现因介入手术操作引起的相关并发症，患者均可耐受该项治疗。

动脉灌注化疗联合靶向药物的综合治疗非小细胞肺癌脑转移，不良反应轻微，适应症广泛，给PS评分较低的患者带来生存获益。随着肿瘤分子病理学的发展、肿瘤基因检测逐渐推广应用，分子靶向药物的研究开发及肿瘤介入治疗的进展，使得更多的晚期NSCLC获益。由于本组病例较少，需要更多的临床研究加以佐证。

## References

[1] Barnholtz-Sloan JS, Sloan AE, Davis FG (2004). Incidence proportions of brain metastases in patients diagnosed (1973 to 2001) in the Metroplitan Detroit Cancer Surveillance System. J Clin Oncol.

[2] Mujoomdar A, Austin JH, Malholtra R (2007). Clinical predictors of metastatic disease to the brain from non-small cell lung carcinoma: primary tumor size, cell type, and lymph node metastases. Radiology.

[3] Kreth FW, Muacevic A, Siefert A (2000). In regard to Dr. Kondzioka et al: stereotactic radiosurgery plus whole brain radiotherapy alone for patiants with multiple brain metastases. Int J Radiat Oncol Biol Phys.

[4] Sundaresan P, Yehiaian-Alvandi R, Gebski V (2010). Prognostic index to identify patients who may not benefit from whole brain radiotherapy for multipile brain metastases from lung cancer. J Med Imaging Radiat Oncol.

[5] Barlesi F, Gervais R, Lena H (2011). Pemetrexed and cisplatin as first-line chemotherapy for advanced non-small cell lung cancer (NSCLC) with asymptomatic inoperable brain metases: a multicenter phase Ⅱ trial (GFPC 07-01). Ann Oncol.

[6] Serizawa T, Hirai T, Nagano O (2010). Gamma knife surgery for 1-10 brain metastases without prophylactic whole-brain radiation therapy: analysis of cases meeting the Japanese prospective multi-institute study (JLGK0901) inclusion criteria. J Neurooncol.

[7] Jiang R, Tian HM, Lv DL (2000). Research of implanted catheter system of super-selective intra-arterial regional arterial infusion involved in the treatment of malignant brain tumor. Jie Ru Fang She Xue Za Zhi.

[8] Yuan JB, Shi JY, Chuo ZH (2005). Efficacy obersavation of interventional treatment of advanced lung cancer with systemic chemotherapy. Shi Yong Zhong Liu Za Zhi.

[9] Liu J, Wang Y, Zhang B (2009). Via bronchial artery interventional therapy lung cancer (report of 206 cases). Xian Dai Yi Yong Ying Xiang Xue.

[10] Zhang L, Ma SL, Song X (2011). Efficacy, tolerability, and biomarker analyses from a phase Ⅲ, randomized, placebo-controlled, parallel group study of gefitinib as maintenance therapy in patients with locally advanced or metastatic non-small cell lung cancer (NSCLC; INFORM; C-TONG 0804). J Clin Oncol.

[11] Zhou C, Wu YL, Chen G (2011). Updated efficacy and quality of life (QoL) analyses in OPTIMAL, a phase Ⅲ, randomized, open-label study of first-line erlotinib vs gemcitabine/carboplatin in patients with EGFR activating-mutation positive (*EGFR* Act Mut^+^) advanced non-small cell lung cancer (NSCLC). J Clin Oncol.

[12] Tan FL, Zhang L, Zhao Q (2009). Pharmacological and clinical evaluation of new drug icotinib. Zhongguo Xin Yao Za Zhi.

[13] Sun Y, Shi Y, Zhang L (2011). A randomized, double-blind phase Ⅲ study of icotinib versus gefitinib in patients with advanced non-small cell lung cancer (NSCLC) previously treated with chemotherapy (ICOGEN). J Clin Oncol (Meeting Abstracts).

